# Mechanical power and 30-day mortality in mechanically ventilated, critically ill patients with and without Coronavirus Disease-2019: a hospital registry study

**DOI:** 10.1186/s40560-023-00662-7

**Published:** 2023-04-06

**Authors:** Basit A. Azizi, Ricardo Munoz-Acuna, Aiman Suleiman, Elena Ahrens, Simone Redaelli, Tim M. Tartler, Guanqing Chen, Boris Jung, Daniel Talmor, Elias N. Baedorf-Kassis, Maximilian S. Schaefer

**Affiliations:** 1 Department of Anesthesia, Critical Care and Pain Medicine, Beth Israel Deaconess Medical Center, Harvard Medical School, Brookline Ave 330, Boston, MA USA; 2 Center for Anesthesia Research Excellence (CARE), Beth Israel Deaconess Medical Center, Harvard Medical School, Boston, MA USA; 3 Division of Pulmonary and Critical Care, Beth Israel Deaconess Medical Center, Harvard Medical School, Boston, MA USA; 4 Department of Anesthesiology, Duesseldorf University Hospital, Duesseldorf, Germany

**Keywords:** COVID-19, Intensive care unit, Respiratory distress syndrome, Respiratory insufficiency, New England, Ventilator-induced lung injury

## Abstract

**Background:**

Previous studies linked a high intensity of ventilation, measured as mechanical power, to mortality in patients suffering from “classic” ARDS. By contrast, mechanically ventilated patients with a diagnosis of COVID-19 may present with intact pulmonary mechanics while undergoing mechanical ventilation for longer periods of time. We investigated whether an association between higher mechanical power and mortality is modified by a diagnosis of COVID-19.

**Methods:**

This retrospective study included critically ill, adult patients who were mechanically ventilated for at least 24 h between March 2020 and December 2021 at a tertiary healthcare facility in Boston, Massachusetts. The primary exposure was median mechanical power during the first 24 h of mechanical ventilation, calculated using a previously validated formula. The primary outcome was 30-day mortality. As co-primary analysis, we investigated whether a diagnosis of COVID-19 modified the primary association. We further investigated the association between mechanical power and days being alive and ventilator free and effect modification of this by a diagnosis of COVID-19. Multivariable logistic regression, effect modification and negative binomial regression analyses adjusted for baseline patient characteristics, severity of disease and in-hospital factors, were applied.

**Results:**

1,737 mechanically ventilated patients were included, 411 (23.7%) suffered from COVID-19. 509 (29.3%) died within 30 days. The median mechanical power during the first 24 h of ventilation was 19.3 [14.6–24.0] J/min in patients with and 13.2 [10.2–18.0] J/min in patients without COVID-19. A higher mechanical power was associated with 30-day mortality (OR_adj_ 1.26 per 1-SD, 7.1J/min increase; 95% CI 1.09–1.46; *p* = 0.002). Effect modification and interaction analysis did not support that this association was modified by a diagnosis of COVID-19 (95% CI, 0.81–1.38; *p*-for-interaction = 0.68). A higher mechanical power was associated with a lower number of days alive and ventilator free until day 28 (IRR_adj_ 0.83 per 7.1 J/min increase; 95% CI 0.75–0.91; *p* < 0.001, adjusted risk difference − 2.7 days per 7.1J/min increase; 95% CI − 4.1 to − 1.3).

**Conclusion:**

A higher mechanical power is associated with elevated 30-day mortality. While patients with COVID-19 received mechanical ventilation with higher mechanical power, this association was independent of a concomitant diagnosis of COVID-19.

**Supplementary Information:**

The online version contains supplementary material available at 10.1186/s40560-023-00662-7.

## Background

During invasive mechanical ventilation, the patient’s lungs are subject to tearing and shearing forces, which can lead to ventilator-associated lung injury [1]. The concept of mechanical power seeks to integrate these forces, exerted through respiratory rate, inspiratory volume and pressure into a single measure, with the goal of estimating the energy affecting the respiratory system. This measure provides potential insight into the risk of ventilator-associated lung injury [2–4].

Previous studies proposed that high mechanical power is associated with higher hospital mortality in critically ill patients undergoing invasive mechanical ventilation for “classic” ARDS [5–8]. The coronavirus disease-2019 (COVID-19) pandemic has challenged intensive care units (ICU) worldwide and had a high impact on mortality in mechanically ventilated patients [9–13]. There is a debate whether pulmonary and respiratory system mechanics of mechanically ventilated patients due to COVID-19 consistently reflect those of patients suffering from “classic” acute respiratory distress syndrome (ARDS) [14–21]. In addition, patients with respiratory failure from COVID-19 often require mechanical ventilation for a longer period of time [14] and consequently may be subjected to higher, cumulative amounts of stress and strain. Previous reports suggested that there was no association between classic independent measures of stress, such as driving pressure, but mechanical power and 28-day mortality in patients with COVID-19 [22]. It is currently unclear whether an association between mechanical power and mortality differs in mechanically ventilated patients due to COVID-19 [14]. We investigated whether a higher mechanical power is associated with mortality in critically ill, mechanically ventilated patients, and whether any association is modified by a diagnosis of COVID-19.

## Methods

### Study design and setting

In this hospital registry study, we analyzed ICU cases at Beth Israel Deaconess Medical Center, an academic tertiary healthcare facility in Boston, Massachusetts, United States of America, between March 2020 and December 2021. Adult patients who underwent invasive, controlled mechanical ventilation for more than 24 h in between March 2020 and December 2021 were screened for inclusion in this study. Data were retrieved from electronic hospital management databases, strictly de-identified, and subsequently merged into a research data repository. All procedures were followed in accordance to the Helsinki Declaration of 1975. This study was reviewed by the institutional review board (IRB) at Beth Israel Lahey Health, which determined that it met the criteria for exempt status (study title “Association between mechanical power and 30-day mortality in mechanically ventilated patients during the COVID-19 pandemic: a hospital registry study”, approved on 13th July 2022, protocol number #2022P000458). The requirement for informed consent was waived. Additional file 1: Digital Content, section S1.1 provides further details related to data sources. This manuscript adheres to the STROBE guidelines and the RECORD statements [23, 24].

### Exposure and outcome measures

The primary exposure was the median mechanical power during the first 24 h of controlled mechanical ventilation, defined as: mechanical power (J/min) = 0.098*RR*V_t_*(PEEP + *½[P*_*plat*_* − PEEP]* + *[P*_*peak*_* − P*_*plat*_*]) (respiratory rate, RR; tidal volume, V*_*t*_*; peak inspiratory pressure, P*_*peak*_*; plateau Pressure, P*_*plat*_*; positive end-expiratory pressure, PEEP)* [4, 25]. Average mechanical power was calculated for the first 24 h of mechanical ventilation. The primary outcome was all-cause mortality within 30 days from the start of invasive ventilation. The co-primary analysis effect modifier was a diagnosis of COVID-19, defined as a confirmed positive severe acute respiratory syndrome coronavirus type 2 polymerase chain reaction test or International Classification of Diseases -10 diagnosis of COVID-19. Additional details on the definition of exposure and outcome are provided in Additional file 1: Digital Content, section S1.3.

### Confounder model

All analyses were adjusted for a priori defined confounders based on literature review and clinical plausibility [22, 25]. These variables included the quarter of the pandemic and patient demographics such as age, sex, body mass index, as well as comorbidities, such as chronic obstructive or restrictive lung diseases and smoking status. Further, the Acute Physiology And Chronic Health Evaluation score [26] incorporating patient age, vital signs such as heart rate and body temperature, as well as sodium, potassium and creatinine levels as well as hematocrit, and the Elixhauser Comorbidity Index [27] incorporating various comorbidities such as renal failure, diabetes, metastatic cancer, and pulmonary circulation disorders were defined as confounding variables. Consistent with the definition of the primary exposure, confounding variables were calculated within the first 24 h of controlled mechanical ventilation. Observations with missing items of the APACHE-II score were imputed (Additional file 1: Digital Content, section S1.4).

Analyses were further adjusted for the administration of opioids, vasopressors, non-depolarizing neuromuscular blocking agent infusion [28, 29], fluid balance, the occurrence of prone positioning, the presence of a high N-terminal prohormone of brain natriuretic peptide and high D-Dimer [30, 31]. Further details related to the confounding model are provided in Additional file 1: Digital Content, section S1.4.

### Primary and co-primary analyses

In the primary analysis, we assessed the association between mechanical power and mortality within 30 days after the start of invasive ventilation using multivariable logistic regression analysis. Conditional on an association between mechanical power and 30-day mortality, we conducted the co-primary analysis, where we assessed a potential effect modification of the primary association by a diagnosis of COVID-19. We first applied interaction analysis followed by an analysis stratified by a diagnosis of COVID-19 [32–34]. Further details related to the co-primary analysis are provided in Additional file 1: Digital Content, section S2.

### Secondary and exploratory analyses

In secondary analyses, we examined the influence of each single parameter utilized for calculation of mechanical power (driving pressure, tidal volume, respiratory rate, and PEEP) by dominance analysis [35]. Previously, three major components of mechanical power have been described [36]—a “static” component from PEEP, a dynamic elastic component that reflects energy applied to expand the lung and chest wall and a dynamic resistive component which reflects energy applied to overcome airway resistance. In an additional dominance analysis, we examined the relative influence of each of the previous components on 30-day mortality and examined potential effect modification by a patient’s COVID-19 status if an association is significant.

Additionally, we investigated the association of mechanical power with being alive at day 28 and the number of ventilator-free days after initiation of mechanical ventilation [37].

To address potential differences in patient's respiratory system mechanics, we reinvestigated our primary association in an exact-matched cohort (1:1, caliper 0.01), based on patients’ baseline static respiratory system compliance, standardized to ideal body weight and determined within the first six hours of mechanical ventilation. Details on all secondary analyses are provided in Additional file 1: Digital Content, section S3.

With an exploratory intent, we conducted a subgroup analysis in patients presenting with a PaO_2_/FiO_2_ ratio < 300 mmHg [38].

Additionally, we investigated the primary and co-primary association with the calculation of mechanical power at day 2 [5], the median mechanical power of the first 72 h of mechanical ventilation, as well as with in-ICU mortality, 7-day, 14-day and 28-day in- and out of hospital mortality.

### Sensitivity analyses

We conducted multiple sensitivity analyses to test the robustness of the primary and co-primary models, which included additional adjustment for (1) pH and partial arterial oxygen pressure to fraction of inspired oxygen ratio (P/F ratio); (2) utilization of inspiratory and expiratory transpulmonary pressure measurements to calculate lung-directed mechanical power [39], when available; (3) sedation and analgesia at day one and day two of mechanical ventilation; (4) high-flow oxygen or non-invasive ventilation therapy prior to mechanical ventilation; (5) a subgroup analysis only in patients receiving infusions of neuromuscular blocking agents (NMBA) at any given time point during their course of mechanical ventilation; (6) normalizing mechanical power to ideal body weight [8]; re-assessment of the primary analysis (7) in a cohort without imputation of missing data for the APACHE-II score and with multiple imputation of all missing confounders; (8) a subgroup analysis in only the first case patient of each patient during the study period; (9) re-evaluation of the primary association in a cohort weighted through propensity score matching; and (10) average treatment effects analysis in a cohort weighted through inverse probability treatment weighting; (11) by excluding patients in the period March to May 2020. Details on all sensitivity analyses are provided in Additional file 1: Digital Content, section S4.

### Statistical analyses

For primary and secondary analyses, multivariable logistic regression analyses, as well as negative binomial regression models were performed. Linear combinations of the main effect and interaction terms were calculated to assess effect modification of the primary association by COVID-19 [32–34, 40]. Adjusted odds ratios (OR_adj_) and 95% confidence intervals (CI) are reported for multivariable logistic regression models, with alpha set to 0.05. Continuous variables were classified into clinically relevant categories. Analyses were performed using Stata (Version 16.0, StataCorp LLC, College Station, Texas, USA) and R Statistical Software (Version 4.1.0, Foundation for Statistical Computing, Vienna, Austria). Power analyses were conducted using G*Power [41]. Further details on our statistical analyses, including assessment of model fit and calibration are provided in Additional file 1: Digital Content, section S1.2.

## Results

### Study cohort and characteristics

**Fig. 1:**
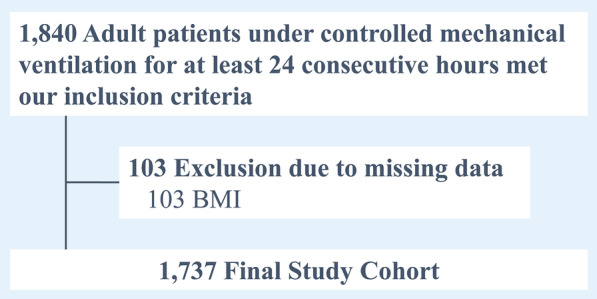
Study flow diagram. *BMI* body mass index. *385 patients with missing APACHE-II scores were imputed

**Fig. 2:**
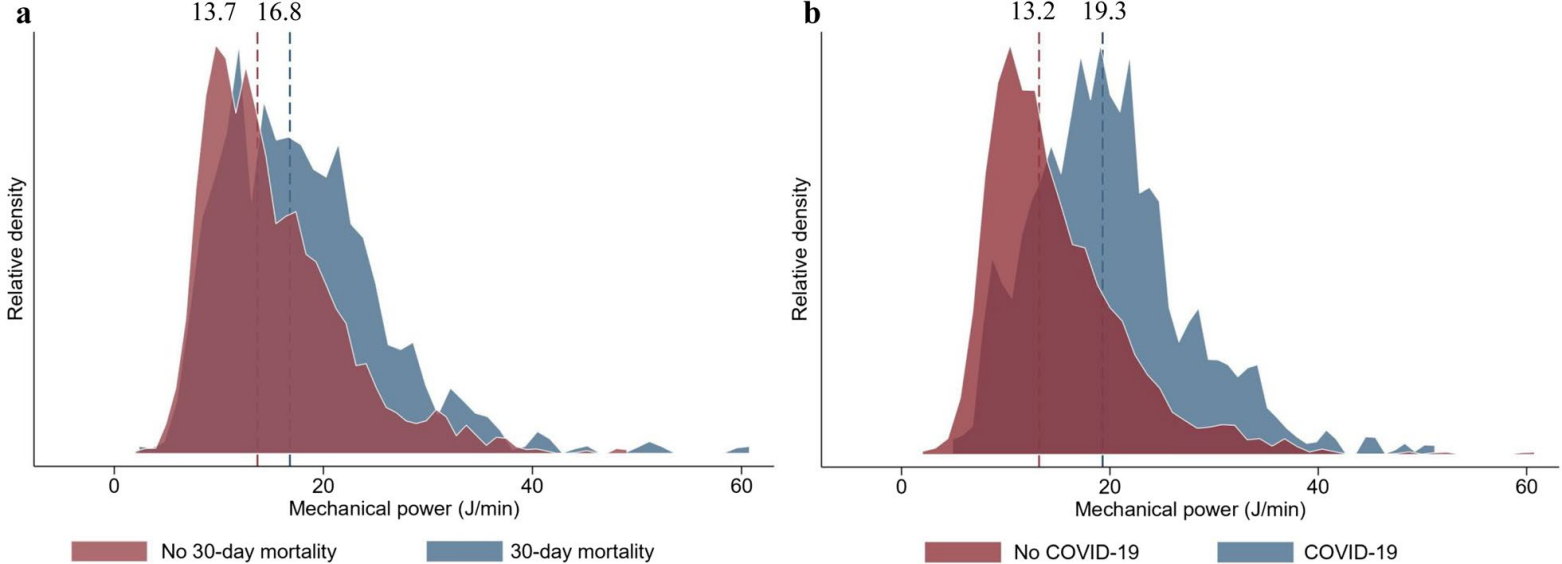
Distribution of mechanical power. Histograms depicting mechanical power distribution between **a** patients with and without 30-day mortality (median [interquartile range] mechanical power in patients who died within 30 days after start of invasive ventilation was 16.8 [12.0–22.3] J/min and in patients who survived 13.7 [10.2–18.6] J/min); and **b** patients with and without Coronavirus Disease 2019 (19.3 [14.6–24.0] J/min in patients with and 13.2 [10.2–18.0] J/min in patients without the disease)

A total of 1,737 patients were included in this study (Fig. 1). Out of these, 509 (29%) patients died within 30 days after the start of invasive ventilation. The median (IQR) mechanical power in the cohort was 14.5 (10.7–19.8) J/min. The distribution of mechanical power is depicted in Fig. 2*.* The median (IQR) duration of mechanical ventilation was 107.2 (53.4–234.6) hours. 411 (23.7%) patients suffered from COVID-19. These patients were ventilated for a median 247 (121–442) hours, compared to 86 (46–173) hours in non-COVID-19 patients. Further details related to patient characteristics and the distribution of variables by COVID-19 diagnosis are provided in Table 1.

**Table 1:** Patient characteristics and distribution of variables by diagnosis of COVID-19

	No diagnosis of COVID-19	Diagnosis of COVID-19	Std. diff
	*N* = 1326	*N* = 411	
Demographics			
Age, years	65 (54–74)	63 (54–72)	0.10
BMI, kg/m^2^	27.6 (23.6–32.6)	30.8 (26.1–36.1)	− 0.37
Sex, female	518 (39.1%)	154 (37.5%)	− 0.03
Comorbidities			
Chronic lung disease	458 (34.5%)	145 (35.3%)	− 0.02
Congestive Heart failure	457 (34.5%)	89 (21.7%)	0.29
Renal failure	360 (27.1%)	96 (23.4%)	0.09
Liver disease	363 (27.4%)	75 (18.2%)	0.22
Diabetes mellitus	451 (34%)	160 (38.9%)	− 0.10
Elixhauser comorbidity score	20 (12–27)	15 (8–23)	0.39
Smoking	686 (51.7%)	163 (39.7%)	0.24
In-hospital factors during first 24 h of mechanical ventilation			
Total duration of mechanical ventilation, hours	97.6 (49.1–215.2)	267.4 (134.5–496.9)	− 0.61
Respiratory rate, 1/min	21 (18–25)	25 (20.5–28)	− 0.64
Positive end-expiratory pressure, cmH_2_O	5 (5–8)	11 (8–14)	− 1.19
Plateau pressure, cmH_2_O	17.5 (15–21)	23.2 (20–27)	− 1.06
Tidal volume, ml/kg IBW	6.4 (6–6.9)	6.1 (5.8–6.6)	0.38
P/F ratio, mmHg	164 (101.3–314)	133.8 (92–204.7)	0.38
Driving pressure, cmH_2_O	11 (9–13)	11.5 (10–14)	− 0.24
Baseline standardized compliance, (ml/kg)/cmH_2_O	0.6 (0.5–0.7)	0.5 (0.4–0.6)	0.19
Baseline standardized elastance, cmH_2_O/(ml/kg)	1.8 (1.4–2.1)	1.9 (1.6–2.4)	− 0.22
Positioned in prone position	26 (2%)	151 (36.7%)	− 0.98
Use of esophageal manometry	80 (6%)	186 (45.3%)	− 1.00
Fluid balance, ml	104 (− 1162–1530.6)	− 784.9 (− 1627.8–268.6)	0.47
Heart rate, 1/min	83 (72–96)	79.5 (69–92)	0.19
Times MAP below 55 mmHg	0 (0–2)	1 (0–5)	− 0.28
Any midazolam administered	298 (22.5%)	177 (43.1%)	− 0.45
Administered propofol, mg	2990.4 (1327.4–4593)	4979 (2965–6905)	− 0.67
Administered vasopressors, mcg/kg norepinephrine equivalents	90.8 (2.4–367.7)	83.8 (19.2–219.6)	0.20
Continuous non-depolarizing NMBA infusion	75 (5.7%)	159 (38.7%)	− 0.87
Administered opioids, mg OME	301.6 (50–577.3)	864.2 (468.2–1206.6)	− 1.07
Arterial pH	7.4 (7.3–7.4)	7.3 (7.3–7.4)	0.21
Partial pressure of arterial CO_2_, mmHg	41 (36–46)	45 (41–52)	− 0.51
D-Dimer > 500 ng/ml	62 (4.7%)	155 (37.7%)	− 0.88
Appearance of elevated NT-proBNP, age adjusted	59 (4.4%)	19 (4.6%)	− 0.01
Creatinine, mg/dL	1.2 (0.8–2.1)	1.2 (0.9–2.1)	0.00
Potassium, mEq/L	4.1 (3.8–4.6)	4.3 (3.9–4.7)	− 0.17
Sodium, mEq/L	138.5 (135–141.5)	138.5 (135–141)	− 0.02
Hematocrit, L/L	30.5 (26.1–36)	34.6 (30.1–39.1)	− 0.48
White blood cells, cells per μL	12.5 (8.7–17.6)	10.5 (7.7–15.4)	0.15
APACHE-II-Score	24 (19–29)	25 (20–28)	− 0.04

Patient characteristics and distribution of variables by the diagnosis of COVID-19

Data are expressed as frequency (prevalence in %), or median (interquartile range [25th–75th percentile])

*BMI* body mass index, *COVID-19* Coronavirus Disease 2019, *IBW* ideal body weight, *P/F ratio* ratio of partial pressure of oxygen in arterial blood and the fraction of oxygen in the inhaled air, *MAP* mean arterial blood pressure, *NMBA* neuromuscular blocking agents, *OME* oral morphine equivalent, *CO2* carbon dioxide, *NT-proBNP* N- terminal prohormone of brain natriuretic peptide, *mEq* milliequivalent; *APACHE-II* Acute Physiology And Chronic Health Evaluation II

### Primary analysis

The mean mechanical power for patients who died versus patients who did not die within 30 days after the start of mechanical ventilation was 15.2 ± 6.7 J/min and 18.0 ± 7.8J/min, respectively. In unadjusted analyses, there was a significant association between a higher mechanical power and 30-day mortality (OR 1.45 per 1-SD increase, 7.1 J/min; 95% CI, 1.31–1.60; *p* < 0.001). After confounder adjustment, these results remained consistent (OR_adj_ 1.26 per 1-SD increase, 7.1J/min; 95% CI, 1.09–1.46; *p* = 0.002), corresponding to an adjusted absolute risk increase of 3.9% (95% CI, 1.4–6.3) per each 7.1 J/min increase.

Among COVID-19 patients, the mean mechanical power was 19.9 ± 7.5 J/min, compared to 14.8 ± 6.5 J/min in the 1,326 (77.3%) patients not suffering from COVID-19. There was no interaction between mechanical power and a diagnosis of COVID-19 with regard to 30-day mortality (95% CI, 0.81–1.38; p-for-interaction = 0.68). Stratified analyses further did not support effect modification (Additional file 1: Digital Content, section S2).

### Secondary and exploratory analyses

**Fig. 3:**
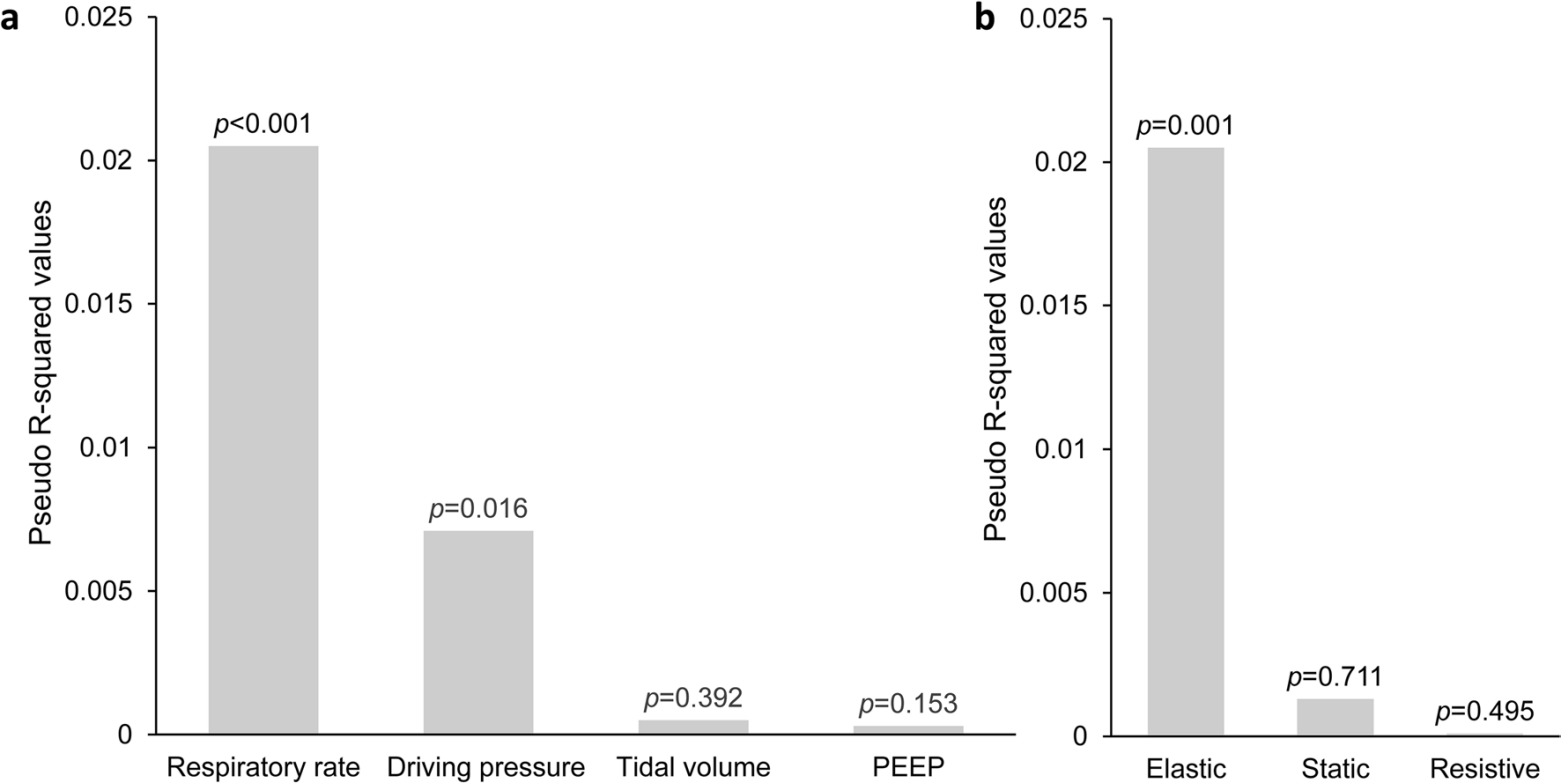
Dominance analyses. Relative dominance of **a** individual parameters of mechanical power and **b** different components of mechanical power with regard to prediction of 30-day mortality. Higher R-squared values depict a higher dominance in predicting 30-day mortality

Compared to tidal volume, PEEP and driving pressure, respiratory rate had the highest contribution to 30-day mortality, followed by driving pressure *(*Fig. 3a*)*. Upon comparing the static, dynamic elastic and dynamic resistive components of mechanical power, the dynamic elastic component was the only significant predictor of mortality (Fig. 3b*)* and this association was not modified by a diagnosis of COVID-19 (95% CI 0.79–1.03; p-for-interaction 0.11).

A higher mechanical power was associated with a lower number of alive and ventilator-free days until day 28 (IRR_adj_ 0.83 per 1-SD increase, 7.1 J/min; 95% CI, 0.75–0.91; *p* < 0.001, adjusted absolute difference − 2.7 days per 7.1J/min increase; 95% CI, − 4.0 to − 1.3, *p* < 0.001). This effect was magnified in COVID-19 patients (IRR_adj_ 0.88 per 1-SD increase, 7.1 J/min; 95% CI, 0.79–0.98; p-for-interaction = 0.008) with an adjusted absolute difference in COVID-19 patients of -3.96 days per 7.1 J/min increase; 95% CI, − 6.19 to − 1.72; and − 1.96 days per 7.1 J/min increase; 95% CI, − 3.56 to − 0.36 in non-COVID-19 patients.

**Fig. 4:**
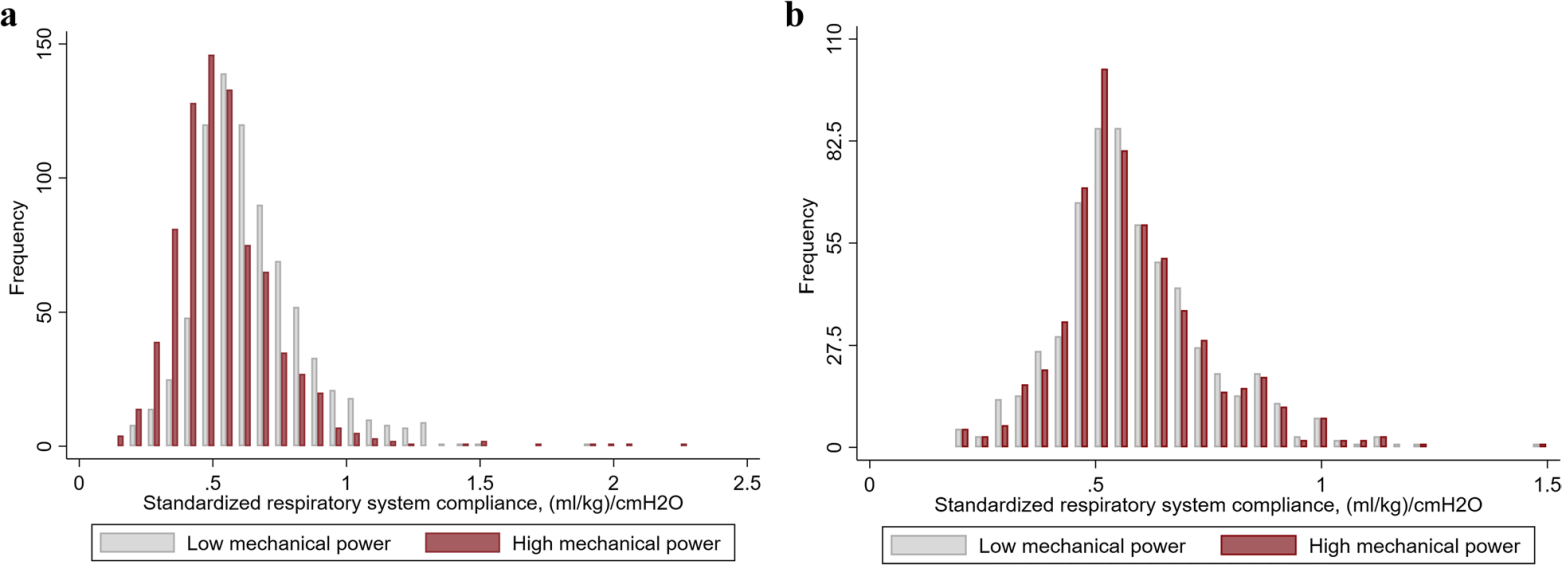
Respiratory system compliance before **a** and after **b** matching. Distribution of standardized respiratory system compliance, defined as the initial respiratory system compliance normalized to ideal body weight, for patients whose lungs were ventilated with low (light grey) versus high (red) median mechanical power before **a** and after **b** matching for respiratory system compliance. Low versus high mechanical power was defined based on the cohort median of 14.5 J/min

Before matching, the median (IQR) baseline standardized static respiratory system compliance was 0.51 (0.42–0.63) (ml/kg)/cmH_2_O and 0.62 (0.51–0.75) (ml/kg)/cmH_2_O (std. diff. = 0.50) for patients receiving high (≥ 14.5 J/min) versus low (< 14.5 J/min) mechanical power, respectively. 1,190 patients were matched. Standardized static respiratory system compliance in the matched cohort was 0.56 (0.49–0.67) (ml/kg)/cmH_2_O and 0.56 (0.48–0.67) (ml/kg)/cmH_2_O (std. diff. = 0.02, Fig. 4) for patients that received high versus low mechanical power. A high mechanical power was associated with a higher risk of 30-day mortality (OR_adj_ 1.44; 95% CI, 1.02–2.04; *p* = 0.038, adjusted risk difference 6.0%; 95% CI, 0.3–11.6). This association was not modified by a diagnosis of COVID-19 (p-for-interaction = 0.55).

Details on our secondary analyses are provided in Additional file 1: Digital Content, section S3.

Among our study cohort, 1,243 (71.5%) patients presented with a PaO_2_/FiO_2_ ratio < 300 mmHg. Analyses in this sub-cohort yielded robust results of the primary (OR_adj_ 1.24 per 1-SD, 7.1J/min increase; 95% CI 1.05–1.46; p = 0.010) and co-primary association (OR_adj_ 1.10 per 1-SD, 7.1J/min increase; 95% CI 0.82–1.47; p-for-interaction = 0.53).

Exploratory analyses revealed a significant association of mechanical power with in-ICU mortality (ORadj 1.33 per 1-SD increase, 7.1 J/min; 95% CI, 1.14–1.55; *p* < 0.001), 7-day mortality (ORadj 1.23 per 1-SD increase, 7.1J/min; 95% CI, 1.03–1.48; *p* = 0.025), 14-day mortality (ORadj 1.25 per 1-SD increase, 7.1 J/min; 95% CI, 1.09–1.49; *p* = 0.002), and 28-day mortality (ORadj 1.27 per 1-SD, 7.1J/min increase; 95% CI 1.10–1.48; *p* = 0.001). Neither of these associations were modified by a diagnosis of COVID-19.

After recalculating mechanical power at day two as well as the first 72 h of mechanical ventilation, the primary results remained robust for the primary and co-primary analysis for day two (ORadj 1.39 per 1-SD, 7.8J/min increase; 95% CI 1.18–1.64; *p* < 0.001 and p-for-interaction = 0.96), as well as for the first 72 h of mechanical ventilation (ORadj 1.21 per 1-SD, 7.5J/min increase; 95% CI 1.02–1.43; *p* = 0.025 and p-for-interaction = 0.38), respectively.

### Sensitivity analyses

The primary associations remained robust throughout all sensitivity analyses. Details on all sensitivity analyses are provided in Additional file 1: Digital Content, section S4.

## Discussion

In this cohort of 1,737 critically ill patients that underwent controlled mechanical ventilation for more than 24 consecutive hours, a higher mechanical power was associated with a higher risk of mortality within 30 days after the start of invasive ventilation. This association was independent of whether patients had a concomitant diagnosis of COVID-19. In addition, a higher mechanical power was associated with a lower number of days alive and ventilator-free, which was slightly more pronounced in patients suffering COVID-19. Among parameters included in mechanical power, the respiratory rate, followed by driving pressure, had the strongest contribution to 30-day mortality. Dissecting mechanical power into static, dynamic elastic and dynamic resistive components revealed that only the dynamic elastic component was significantly associated with mortality both in patients with and without COVID-19.

Our study corroborates and extends the findings of a previous post hoc analysis [5] as well as a retrospective study [22] reporting that higher mechanical power was associated with increased 28-day mortality in patients with respiratory failure due to COVID-19. In our study, mechanical power predicted mortality in both COVID-19 and non-COVID-19 patients, which suggests that the development of ventilator induced lung injury through mechanical power is applicable in patients independent of this etiology of respiratory failure.

Previously, different phenotypes of COVID-19 were proposed [18] and ventilation with low PEEP in many COVID-19 patients was suggested [19]. In addition, respiratory failure during COVID-19 has been proposed to be driven by ventilation–perfusion mismatching, which would be independent from patients’ pulmonary mechanics [19, 42]. By contrast, our study supports previous reports on similar respiratory system mechanics between COVID-19 and non-COVID-19 patients [21, 43] and that a concept of high-intensity mechanical ventilation is associated with mortality in both patient populations.

Our findings corroborate previous studies [20, 44–48] as we found that the dynamic elastic component, mainly driven by driving pressure, was the only predictive component when compared to the dynamic resistive and static components (the component provided in the equation by PEEP). When comparing the individual parameters of mechanical power, respiratory rate had an even bigger contribution on predicting mortality than driving pressure, while it received less attention in literature [49, 50]. This may be in part attributed to the consistent application of lung protective ventilation which heavily focuses on driving pressure as a clinical target, but our findings also support the need for a concept that integrates respiratory rate in addition of stress and strain per breath.

PEEP may have variable impact depending on the individual patient, resulting in overdistension if set too high, or lung collapse if set too low [51]. The use of PEEP within the mechanical power calculation is controversial [36]. While very high levels of PEEP can promote lung injury due to higher stress and strain (which may also increase the dynamic component of mechanical power secondary to increased driving pressure), many patients require higher levels of PEEP to optimize mechanics through avoidance of lung collapse. Our data corroborate these critiques as we could not find any association between the PEEP-driven mechanical power component and 30-day mortality.

It remains unclear whether mechanical power is a marker for illness severity and impaired lung mechanics or a modifiable parameter to target during clinical care [36]. We noted, however, that both high and low mechanical power could occur in patients with poor baseline respiratory system compliance depending on variability in clinical care. We reinvestigated our primary association in a cohort matched by baseline respiratory system compliance calculated from ventilatory parameters after intubation. High mechanical power was associated with a higher risk of 30-day mortality, even after exact matching for baseline dynamic respiratory system compliance.

Recent data from our group suggest that mechanical power is modifiable with changes in tidal volumes and respiratory rate resulting in changes in resulting power with variable impact based upon lung mechanics [25, 52]. This compliance matching, while not directly proving causality or that power is modifiable, suggests that power is more than a simple marker for disease severity and that the association between 30-day mortality and mechanical power is not influenced by COVID-19, regardless of the patient's respiratory system compliance.

Based on our findings, physicians should pay attention to mechanically ventilated patients in the ICU receiving high mechanical power, especially when adjusting respiratory rate and driving pressure. Our study supports clinical practice of lung protective ventilation aimed at lowering the driving pressure and tidal volume, however, based on our findings, clinicians should pay attention also when increasing respiratory rate. Indeed, reducing respiratory rate might be an important step to lower the applied mechanical power, which could be achieved by tolerating permissive hypercapnia. The risks/benefits of such a practice remain beyond the scope of this paper, however, and future prospective studies are warranted to confirm our observations.

### Limitations

The retrospective study design could be susceptible to bias that might confound the results. A causal relationship between high mechanical power and mortality cannot be established. Limitations might as well arise from the definition of COVID-19 due to missing data on outpatient tests and previous, potentially unidentified COVID-19 infections. Limitations might as well arise from the definition of COVID-19 due to missing data on outpatient tests and previous, potentially unidentified COVID-19 infections. In addition, this was a single-center study in an academic health care network in New England, and our findings should be investigated in different hospital settings and geographical locations. However, our observations reflect investigations by others [22]. In addition, the situation of limited resources especially during the initial phases of the pandemic does not reflect current situations in intensive care units in most countries. However, a sensitivity analysis excluding this specific period yielded robust results. In addition, information whether a patient was admitted intubated and how long they were ventilated before admission to the ICU is unknown. However, we performed several sensitivity analyses, including propensity score analyses with inverse treatment probability weighting and generalized propensity score matching to minimize potential bias. Further, highly granular data on vital signs and laboratory values allowed us to adjust for the APACHE-II score, an established risk assessment tool for ICU mortality. Our strength points rely on the adjustment of our model to strong predictors of mortality in COVID-19, such as D-Dimer and NT-proBNP. Thus, we believe that our study helps inform clinicians and contributes to the design of future randomized controlled trials investigating the association between mechanical power and mortality in critically ill patients.

## Conclusion

A higher mechanical power is associated with an increased risk of 30-day mortality and lower days alive and ventilator free in critically ill patients. These findings did not differ between COVID-19 and non-COVID-19 patients. We identified the respiratory rate and driving pressure as the key drivers of the association between mechanical power and mortality. Physicians should carefully adjust these parameters to ensure adequate mechanical ventilation in patients with and without COVID-19.

## Supplementary Information

**Additional file 1.** Additional details on methods and results.

## Data Availability

The dataset used is available upon a justified request.

## Abbreviations

ICU: Intensive care unit
ARDS: Acute respiratory distress syndrome
COVID-19: Coronavirus disease 2019
RR: Respiratory rate
V_t_: Tidal volume
PIP: Peak inspiratory pressure
P_plat_: Plateau pressure
PEEP: Positive end-expiratory pressure
APACHE-II: Acute Physiology and Chronic Health Evaluation
NT-proBNP: N-terminal prohormone of brain natriuretic peptide
P/F-ratio: Partial arterial oxygen pressure to fraction of inspired oxygen ratio
NMBA: Neuromuscular blocking agents
OR_adj_: Adjusted odds ratios
CI: Confidence intervals
IRR_adj_: Adjusted incidence rate ratios
SD: Standard deviation
IQR: Interquartile range
AU-ROC: Area under the receiver operating characteristic
