# Supplementary material for: Mechanical power and 30-day mortality in mechanically ventilated, critically ill patients with and without Coronavirus Disease-2019: a hospital registry study
Source: J Intensive Care. 2023 Apr 6;11:14. doi: 10.1186/s40560-023-00662-7 (PMC10077655; doi:10.1186/s40560-023-00662-7)
Supplement: Supplementary file 1 — Additional file 1. Additional details on methods and results. [file 40560_2023_662_MOESM1_ESM.docx]

**Mechanical power and 30-day mortality in mechanically ventilated, critically ill patients with and without Coronavirus Disease-2019: A hospital registry study.**

Basit A. Azizi, cand. med.^1,2^, Ricardo Munoz-Acuna, MD^1,2^, Aiman Suleiman, MD, MSc^1,2,^, Elena Ahrens, cand. med.^1,2^, Simone Redaelli, MD^1,2^, Tim M. Tartler, cand. med.^1,2^, Guanqing Chen, PhD^2^, Boris Jung, MD, PhD^1,2^, Daniel Talmor, MD, MPH^1^, Elias N. Baedorf-Kassis, MD^3#^, Maximilian S. Schaefer, MD, PhD^1,2#^

*^#^These authors contributed equally.*

*^1^Department of Anesthesia, Critical Care and Pain Medicine, Beth Israel Deaconess Medical Center, Harvard Medical School, Boston, MA, USA.*

*^2^Center for Anesthesia Research Excellence (CARE), Beth Israel Deaconess Medical Center, Harvard Medical School, Boston, MA, USA.*

*^3^Division of Pulmonary and Critical Care Beth Israel Deaconess Medical Center, Harvard Medical School, Boston, MA, USA.*

*To respect space limitations in the main manuscript, we present detailed description of methods and analyses including the secondary and sensitivity analyses.*

Additional file 1

**Table of contents**

APPENDIX S1: Additional METHODS

S1.1 Data sources 4

S1.2 Statistical analyses 4

S1.3 Exposure and outcome variables 5

S1.4 Potential confounding variables 6

APPENDIX S2: CO-PRIMARY ANALYSIS 7

APPENDIX S3: SECONDARY ANALYSES

S.3.1 Dominance analyses 8

S.3.2 Ventilator-free days and alive until day 28 8

S.3.3 Matching by patients’ compliance 9

APPENDIX S4: EXPLORATORY ANALYSES

S.4.1 Miscellaneous outcome definitions 9

S.4.2 Recalculation of mechanical power at day two of mechanical ventilation 9

S.4.3 Recalculation of mechanical power at the first 72 hours of mechanical ventilation 9

APPENDIX S5: SENSITIVITY ANALYSES

S.4.1 Adjustment for P/F ratio and pH 10

S.4.2 Adjustment of mechanical power to inspiratory and expiratory transpulmonary pressure measurements 10

S.4.3 Adjustment of mechanical power to sedation and analgesia at day one and day

two of mechanical ventilation 10

S.4.4 Adjustment of mechanical power to high flow oxygen therapy and non-invasive ventilation prior to mechanical ventilation 11

S.4.5 Subgroup analysis in patients receiving continuous infusions of neuromuscular blocking agents 11

S.4.6 Normalization of mechanical power to ideal body weight 11

S.4.7 Primary analysis with and without multiple imputation 11

S.4.8 Primary model only in first patient cases 12

S.4.9 Propensity score matching 12

S.4.10 Inverse probability treatment weighting 12

S.4.11 Exclusion of patients in the period of March to May 2020 13

APPENDIX S5: SUPPLEMENTAL FIGURES AND TABLES

Figure S1. Receiver Operating Characteristic (ROC) curve of the primary regression model. 14

Figure S2. Calibration plot of the primary regression model 15

Table S1. Patient characteristics and distribution of variables by high and low mechanical power 16

Table S2. Absolute number and percentage of missing values for covariates in our study cohort 19

REFERENCES 20

# APPENDIX S1: SUPPLEMENTAL METHODS

## S1.1 Data sources

Data used for this study were collected at Beth Israel Deaconess Medical Center (BIDMC), a tertiary healthcare network in Boston, Massachusetts, USA. The data were obtained from the institutional Intensive Care Research Data Repository (ICRDR), which combines de-identified intensive care related patient data from MetaVision, laboratory registry (LAB), the billing registry Casemix and the Admission Discharge Transfer (ADT) database. Further, data were collected from the institutional Research Data Repository (RDR), which combines de-identified information from AIMS, Perioperative Information Management System (PIMS), Casemix, and ADT. Demographics, admission, and discharge information were obtained from both ADT and Casemix. Data were also complemented with diagnostic codes billed within the healthcare network from Casemix. All patient data were strictly de-identified and merged into a single combined dataset. This manuscript adheres to the Strengthening the Reporting of Observational Studies in Epidemiology (STROBE) guidelines and the REporting of studies Conducted using Observational Routinely-collected Data (RECORD) statements [1,2].

## S1.2 Statistical analyses

The statistical analyses, study endpoints, and confounding variables with the exception of exploratory analyses were defined *a priori*. Adjusted incidence rate ratios (IRR_adj_) are reported for negative binomial regression models. The primary regression model was applied and evaluated with forced variable entry to ensure that estimates could be interpreted in a conventional manner. The correlation and potential multicollinearity between confounding variables and the primary exposure were analyzed by using the estimation of Pearson's correlation coefficient and the variance inflation factor [3]. Model calibration was evaluated and indicated an acceptable model fit. Model discrimination was assessed using the concordance c-statistic, which in our case corresponds to an acceptable area under the receiver operating characteristic (AU-ROC) curve at 0.76 (Figure S1) [4]. A previous study reported an incidence of 30-day mortality in mechanically ventilated patients of 30% [5]. Assuming a two-sided alpha of 0.05 and the aforementioned baseline risk of 30-day mortality, the sample size of 1,737 patients included in this study provided a power of 98.8% to detect a clinically significant difference in the risk of 30-day mortality of at least 15.0% between patients who were exposed to high mechanical power, and those who were not. Power analysis was conducted using G*Power (Version 3.1.9.4) [6].

## S1.3 Exposure and outcome variables

Classical physics describes the principle of power, which reflects work over time. The application of force over a certain distance results in work, measured in joules. If this is now transferred to a three-dimensional context, namely the lung, distance and force are reflected by volume and pressure, respectively. The area under the curve of the pressure-volume loop resembles work. The mechanical power equation [7] can be simplified derived by explaining its individual components: (1) The conversion factor for units of pressure (cmH_2_O and Pascal) is *0.098*; (2) the time component of the classical work-power derivation is reflected by the respiratory rate; (3) the distance travelled per breath is depicted by tidal volume; (4) The static component (*0.098*RR*V_t_*PEEP*), the dynamic elastic component (*0.098*RR*V_t_*(½[P_plat_−PEEP]*) and the dynamic resistance component (*0.098*RR*V_t_*[P_peak_−P_plat_]) (respiratory rate, RR; tidal volume, V_t_; peak inspiratory pressure, P_peak_; plateau Pressure, P_plat_; positive end-expiratory pressure, PEEP)* are grouped together as the pressure-dependent component and are summarized in the area under the curve in the pressure-volume loop.

The primary exposure was the median mechanical power calculated over the duration of mechanical ventilation during the first 24 hours on controlled ventilator settings, defined as pressure or volume-controlled modes. Results are reported per each standard deviation increment increase in mechanical power, corresponding to 7.1 J/min per increment. Only data validated by a respiratory therapist or physician were used in this study. In 84 (4.8%) patients with missing values for plateau pressure, the peak inspiratory pressure was used as a substitute [8]. COVID-19 was defined based on a confirmed positive severe acute respiratory syndrome coronavirus type 2 (SARS-CoV-2) polymerase chain reaction test (PCR), up to 4 days pre-admission and 14 days post-admission, or International Classification of Diseases (ICD)-10 diagnosis of COVID-19, coded as U07.1 billed with the index stay in the ICU. Data on mortality were collected from the national death register through the hospital network.

## S1.4 Potential confounding variables

According to the linearity assumption, confounding variables were categorized into quintiles (age, vasopressors, fluids, opioids, APACHE-II score [9] (incorporating patient age, vital signs such as heart rate and body temperature, as well as laboratory values including sodium, potassium, creatinine and hematocrit), and the Elixhauser Comorbidity Index [10] (incorporating various comorbidities such as renal failure, diabetes, metastatic cancer, and pulmonary circulation disorders), or clinically relevant categories (body mass index). Sex, smoking, chronic lung diseases, positive or negative fluidbalance, the occurrence of a high D-Dimer or high N-terminal prohormone of brain natriuretic peptide (NT-proBNP), the administration of ketamine, dexmedetomidine, midazolam and non-depolarizing neuro-muscular blocking agents (NMBA) were included as binary variables. The injection of NMBA was defined as the administration at any given time point after one hour of mechanical ventilation start, excluding boluses. Opioid doses were calculated in oral morphine equivalent doses. Vasopressor doses were calculated in norepinephrine equivalents. The APACHE-II score was calculated based on 12 physiological and two disease-related components. If one of those components was missing (*Table S2)*, the whole score was set to missing. Therefore, we imputed missing APACHE-II values prior to analysis using multiple imputation by chained equations in R Statistical Software (Version 4.2.0, Foundation for Statistical Computing, Vienna, Austria). Elevated NT-proBNP levels were defined by the age-cut points <50, 50-75, and >75, with corresponding NT-proBNP levels 450, 900 and 1800 pg/mL, respectively and used as a binary variable [11]. High D-Dimer levels were defined as D-Dimer >500 ng/ml and used as a binary variable [12]_._

# APPENDIX S2: CO-PRIMARY ANALYSIS

Our co-primary hypothesis was that any association between mechanical power and mortality was modified by a diagnosis of COVID-19. To assess our hypothesis, we conducted an interaction term analysis as it is popular in practice to assess the existence of effect modifier [13–18]*.* As effect modification and interaction do not always coincide, we conducted a crude analysis (adjustment for the primary confounder model but not COVID-19) and stratified the analysis by patients' COVID-19 status to examine whether the effect of mechanical power is heterogeneous and modified by a COVID-19 diagnosis [19]. These results show that overall, mechanical power is associated with mortality (OR_adj_ 1.25 per 1-SD, 7.1 J/min increase; 95% CI 1.08-1.45; p=0.003), as well as in the subgroups of patients with (OR_adj_ 1.44 per 1-SD, 7.1 J/min increase; 95% CI 1.06-1.94; p=0.019) and without (OR_adj_ 1.24 per 1-SD, 7.1 J/min increase; 95% CI 1.04-1.47; p=0.018) a diagnosis of COVID-19. As shown, the ORadj for the non-COVID-19 patients is close to the crude OR_adj_ while there is only a marginal difference in the OR_adj_ between COVID-19 and non-COVID-19 patients. In conjunction with our interaction term analysis, we do not assume effect modification for this analysis.

# APPENDIX S3: SECONDARY ANALYSES

## S.3.1 Dominance analyses

The relative influence of each component of mechanical power (driving pressure, tidal volume, respiratory rate, and PEEP, as well as the static, dynamic elastic and dynamic resistive components) in predicting 30-day mortality was examined through dominance analyses in each predictor group. In this analysis, a predictor was considered dominant if it had a greater influence on 30-day mortality compared to the other predictors based on pseudo-r-squared values. When dissecting mechanical power into respiratory rate, tidal volume, PEEP and driving pressure, the respiratory rate had the highest contribution to 30-day mortality (pseudo r^2^ = 0.0205; p<0.001), followed by driving pressure (pseudo-r^2^ = 0.071; p=0.016). PEEP and tidal volume had no significant dominance on mortality (p =0.392 p=0.153, respectively). The elastic component had the highest and only significant influence on 30-day mortality when compared to the static and resistive components of mechanical power (pseudo r^2^ = 0.0206; p=0.001).

## S.3.2 Ventilator-free days and alive until day 28

We investigated the effect of mechanical power on 28-day mortality and the amount of ventilator free days, as previously described in other studies [20]. During their ICU stay patients may be extubated and free from mechanical ventilation and then being intubated due to worsening of their condition. Those periods free from invasive ventilation, if lasting for at least 24 consecutive hours in patients with multiple extubation and re-intubation were calculated and summarized. Patients who did not survive until day 28 or received invasive ventilation for more than 28 days were considered to have zero ventilator-free days.

## S.3.3 Matching by patients’ compliance

To address potential differences in the patient's respiratory system mechanics, we conducted an exact matching based on the initial static respiratory system compliance, standardized to the ideal body weight, using a 1:1 scheme and a caliper of 0.01 (ml/kg)/cmH_2_O. The ideal body weight was calculated based on the formula: Ideal body weight=50+2.3*((height (in metre)*39.3701)-60) for male sex and: Ideal body weight=45.5+2.3*((height (in metre)*39.3701)-60) for female sex. 148 observations with missing initial data, defined as data during the first 6h of mechanical ventilation, were dropped.

# APPENDIX S4: SENSITIVITY ANALYSES

## S.4.1 Adjustment for P/F ratio and pH

We reperformed our primary model with additional confounding adjustment for arterial pH and P/F-ratio, since these measurements are already part of the APACHE-II score, but have a high clinical relevance, and our results stayed consistent, with MP significantly associated with 30-day mortality (OR_adj_ 1.20 per 1-SD increase, 7.2 J/min; 95% CI, 1.03-1.39; p=0.022).

## S.4.2 Adjustment of mechanical power to inspiratory and expiratory transpulmonary pressure measurements

The setting of PEEP and plateau pressure are better adjusted to the patient through transpulmonary pressure manometry. Therefore, we used the inspiratory and expiratory transpulmonary pressure measurements as surrogates for plateau pressure and PEEP to rule out potential bias arising from better health care and monitoring provided in those patients [21]. The association with 30-day mortality remained robust (n=266; OR_adj_ 1.22 per 1-SD increase, 7.6 J/min; 95% CI, 1.04-1.44; p=0.017).

## S.4.3 Adjustment of mechanical power to sedation and analgesia at day one and two of mechanical ventilation

We conducted an additional sensitivity analysis, adjusting for sedation and analgesia administration, including ketamine, midazolam, dexmedetomidine during controlled mechanical ventilation. We conducted additional confounding at day one of mechanical ventilation (ORadj 1.26 per 1-SD, 7.1 J/min increase; 95% CI 1.08-1.46; p=0.002 and p-for-interaction=0.72), as well as for day 2 of mechanical ventilation (ORadj 1.36 per 1-SD, 7.1 J/min increase; 95% CI 1.15-1.61; p<0.001 and p-for-interaction=0.97), respectively.

## S.4.4 Adjustment of mechanical power to high flow oxygen therapy or non-invasive ventilation prior to mechanical ventilation

We conducted an additional sensitivity analysis, adjusting for duration of high-flow oxygen therapy or non-invasive mechanical ventilation before study inclusion within the same ICU stay. 271 (15.6%) patients have been non-invasively ventilated prior to start of mechanical ventilation. The median (IQR) time of high flow or non-invasive mechanical ventilation prior to invasive mechanical ventilation was 1 (1-1) day. The primary and co-primary results remained robust (ORadj 1.21 per 1-SD, 7.1 J/min increase; 95% CI 1.05-1.41; p=0.007 and p-for-interaction=0.72, respectively).

## S.4.5 Subgroup analysis in patients receiving continuous infusions of neuromuscular blocking agents

After exclusion of 1,504 patients without continuous administration of NMBA, defined as the administration of NMBA after the first hour from intubation without boluses, the association between mechanical power and 30-day mortality remained robust (OR_adj_ 1.60 per 1-SD increase, 8.6 J/min; 95% CI, 1.01-2.53; p=0.045).

## S.4.6 Normalization of mechanical power to ideal body weight

To further achieve better generalizability of our findings, we normalized mechanical power to ideal body weight as proposed by Zhang et al. [22] by dividing mechanical power by the ideal body weight using the former described formula. The association with 30-day mortality remained robust (OR_adj_ 1.29 per 1-SD increase, ~110*10^-3^ J/min/kg; 95% CI, 1.11-1.51; p=0.001).

## S.4.7 Primary analysis with and without multiple imputation

Our primary findings were confirmed in the cohort without imputation of APACHE-II score (n=1,383; OR_adj_ 1.18 per 1-SD increase, 7.1 J/min; 95% CI, 1.00-1.39; p=0.041) as well as in the cohort with multiple imputations of all missing variables (n=1,840; OR_adj_ 1.20 per 1-SD increase, 7.1 J/min; 95% CI, 1.04-1.39; p=0.011).

## S.4.8 Primary model only in first patient cases

A patient can be admitted and mechanically ventilated multiple times during our study period which can therefore inflate the probability of having the outcome. A subgroup analysis keeping each patient's first case during the study period was conducted and our primary findings were confirmed (n=1,653; OR_adj_ 1.27 per 1-SD increase, 7.1 J/min; 95% CI, 1.09-1.48; p=0.001).

## S.4.9 Propensity score matching

We investigated the primary model in a cohort weighted through propensity score matching to account for imbalances between patients who received high (≥14.5 J/min) versus low (<14.5 J/min) mechanical power. The likelihood of a patient to receive high mechanical power was estimated through calculating a propensity-score adjusted for the confounders used in the multivariate logistic regression model. We identified matched pairs by using a non-replacement algorithm within a closeness range of 0.00001, then 0.0001, etc. up to a closeness range of 0.01 of the propensity score, using a 1:1 scheme. Effectiveness of matching was assessed by calculating weighted standardized differences of confounders after propensity-score matching. 595 patients receiving low mechanical power were matched to 595 patients who received high mechanical power. Analysis in the matched cohort yielded consistent results (OR_adj_ 1.44; 95% CI, 1.02-2.04; p=0.038).

## S.4.10 Inverse probability treatment weighting

We investigated the average treatment effects (ATE) and mean potential-outcome coefficients (POE) in a cohort weighted through inverse probability treatment weighting which confirmed our primary findings (ATE coefficient 0.12; 95% CI, 0.07-0.17; p<0.001; POE coefficient 0.22; 95% CI, 0.19-0.26; p<0.001).

## S.4.11 Exclusion of patients in the period March to May 2020

To address resource limitation at the beginning of the pandemic, we have added another sensitivity analysis excluding the period March to May 2020. This analysis yielded robust results (ORadj 1.27 per 1-SD, 7.1 J/min increase; 95% CI 1.07-1.50; p=0.006 and p-for-interaction=0.99).

# APPENDIX S5: SUPPLEMENTAL FIGURES AND TABLES

#
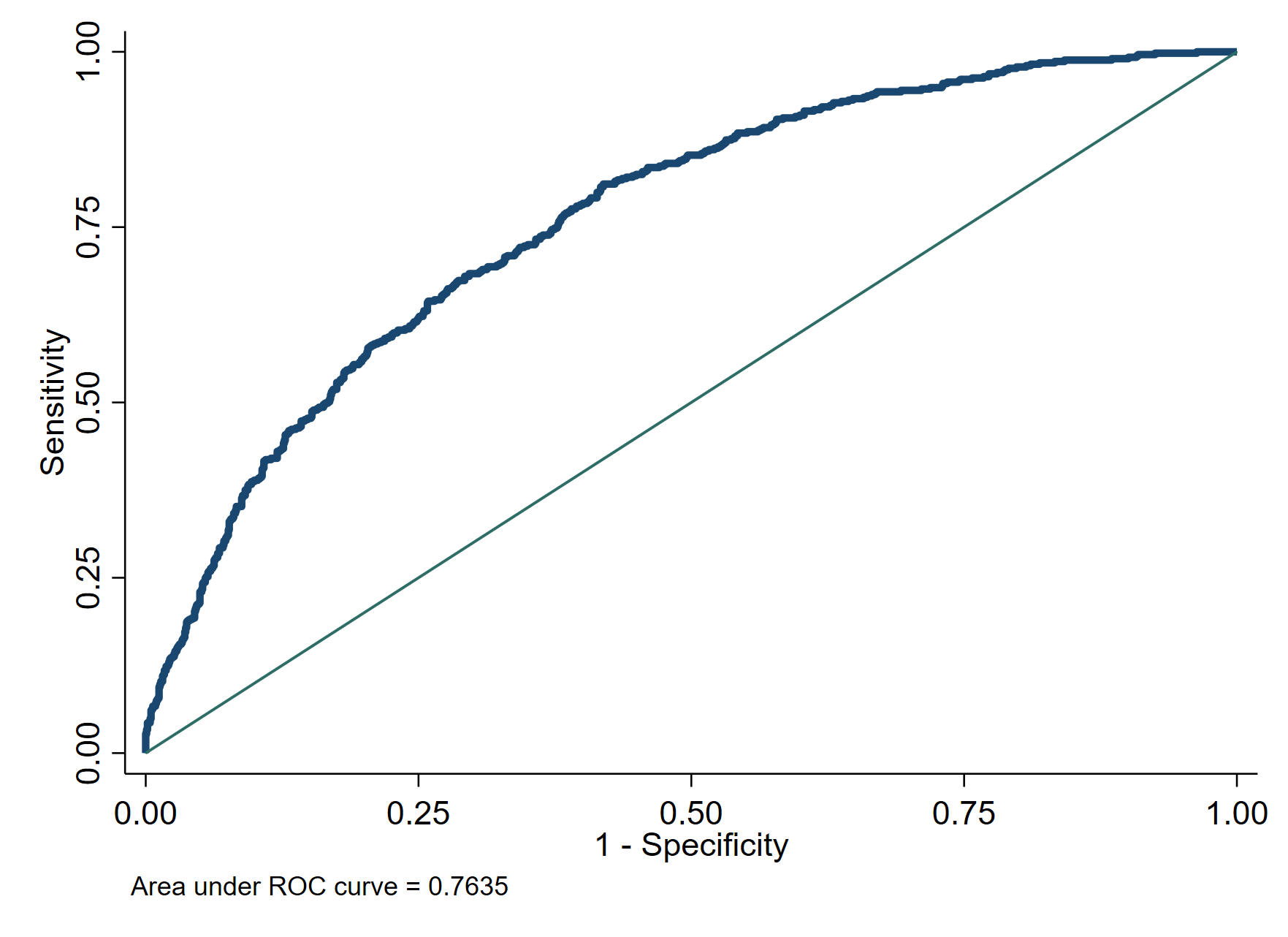


## Figure S1. Receiver Operating Characteristic (ROC) curve of the primary regression model.


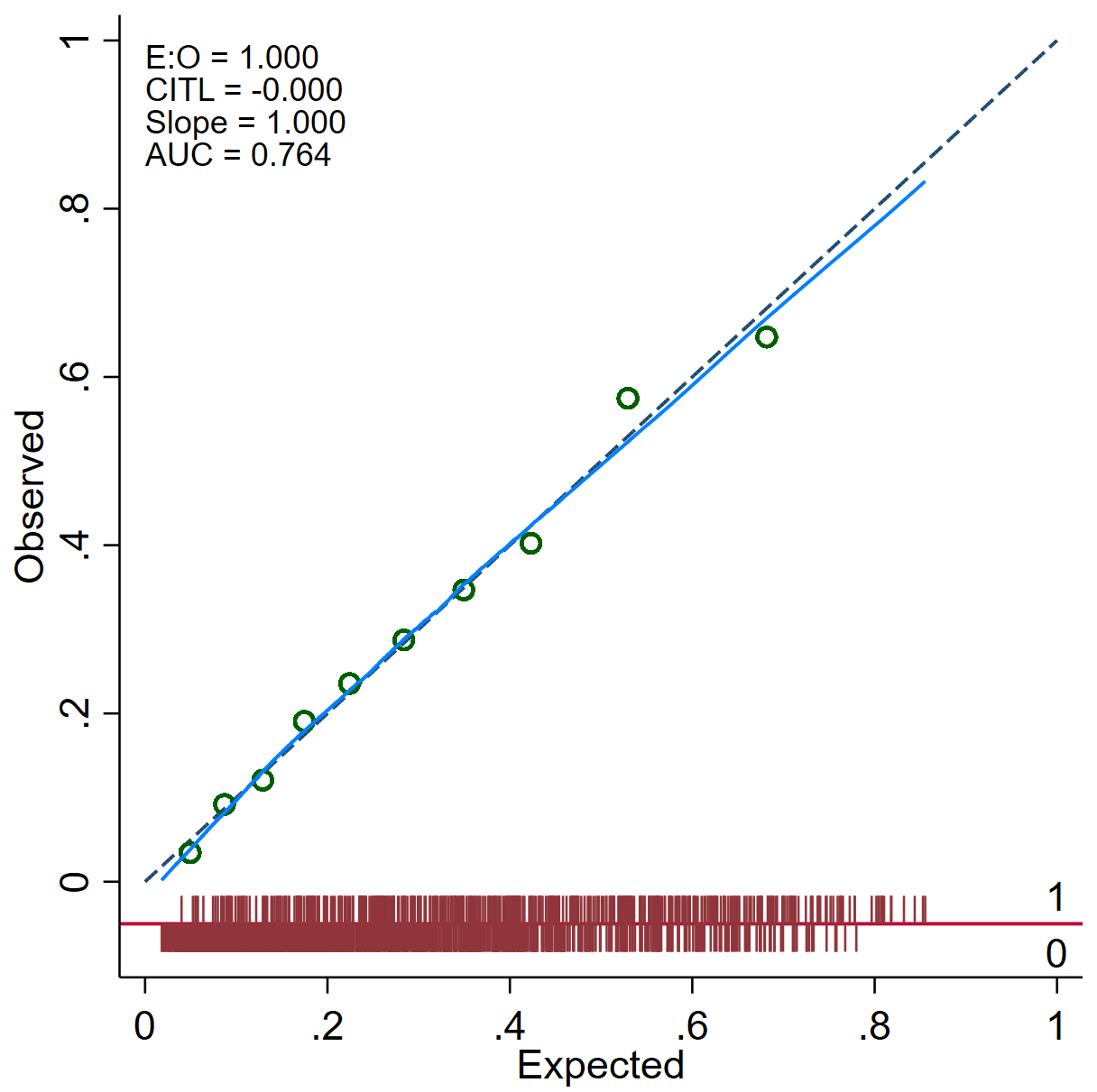


## Figure S2. Calibration plot of the primary regression model.

Each dot represents a decile of the predicted probability of 30-day mortality.

E:O: Ratio of expected and observed outcomes; AUC: Area under the curve; CITL: Calibration-In-The-Large.

## Table 1. Patient characteristics and distribution of variables by high and low mechanical power

|  | **Low mechanical power (<14.5 J/min)** | **High mechanical power (≥14.5 J/min)** | **Std. diff** |
| --- | --- | --- | --- |
|  | **N=870** | **N=867** |  |
| **Demographics** |  |  |  |
| Age, years | 67 (55 - 76) | 63 (53 - 71) | 0.20 |
| BMI, kg/m^2^ | 26.5 (23.0 - 30.6) | 30.9 (25.9 - 36.6) | -0.63 |
| Sex, female | 395 (45.4%) | 277 (31.9%) | -0.28 |
| **Comorbidities** |  |  |  |
| COVID-19 | 101 (11.6%) | 310 (35.8%) | -0.59 |
| Chronic lung disease | 271 (31.1%) | 332 (38.3%) | -0.15 |
| Congestive Heart Failure | 271 (31.1%) | 275 (31.7%) | -0.01 |
| Renal Failure | 225 (25.9%) | 231 (26.6%) | -0.02 |
| Liver Disease | 193 (22.2%) | 245 (28.3%) | -0.14 |
| Diabetes mellitus | 300 (34.5%) | 311 (35.9%) | -0.03 |
| Elixhauser comorbidity score | 19 (11 - 26) | 19 (11 - 27) | 0.00 |
| Smoking | 389 (44.7%) | 460 (53.1%) | -0.17 |
| **In-hospital factors during first 24 hours of mechanical ventilation** |  |  |  |
| Respiratory rate, 1/min | 19.0 (16.5 - 21.5) | 26.0 (22.0 - 28.0) | -1.56 |
| Positive end-expiratory pressure, cmH_2_O | 5 (5 - 5) | 10 (8 - 12) | -1.43 |
| Plateau pressure, cmH_2_O | 16.0 (14.0 - 18.0) | 22.0 (19.0 - 25.3) | -1.52 |
| Tidal volume, ml/kg IBW | 6.3 (6.0 - 6.8) | 6.3 (6.0 - 6.8) | 0.08 |
| P/F ratio, mmHg | 205.0 (112.5 - 377.5) | 133.9 (89.5 - 211.8) | 0.56 |
| Driving pressure, cmH_2_O | 10.0 (8.5 - 12.0) | 12.0 (10.0 - 14.0) | -0.57 |
| Baseline standardized compliance, (ml/kg)/cmH_2_O | 0.6 (0.5 - 0.7) | 0.5 (0.4 - 0.6) | 0.49 |
| Baseline standardized elastance, cmH_2_O/(ml/kg) | 1.6 (1.3 - 2.0) | 2.0 (1.6 - 2.4) | -0.53 |
| Positioned in prone position | 13 (1.5%) | 164 (18.9%) | -0.60 |
| Use of esophageal manometry | 18 (2.1%) | 248 (28.6%) | -0.79 |
| Fluid balance, ml | 125.2 (-994.1 - 1452.8) | -395.4 (-1602.6 - 966.5) | 0.26 |
| Heart rate, 1/min | 80.0 (70.0 - 93.0) | 84.0 (72.5 - 97.5) | -0.19 |
| Times MAP below 55 mmHg | 0 (0 - 1) | 1 (0 - 4) | -0.34 |
| Any midazolam administered | 171 (19.7%) | 304 (35.1%) | -0.35 |
| Administered propofol, mg | 2810. (1117.8 - 4107.8) | 3998.0 (2160.0 - 5986.0) | -0.56 |
| Administered vasopressors, mcg/kg norepinephrine equivalents | 50.0 (0.0 - 203.6) | 134.3 (30.8 - 505.1) | -0.39 |
| Continuous non-depolarizing NMBA infusion | 28 (3.2%) | 206 (23.8%) | -0.63 |
| Administered opioids, mg OME | 259.4 (31.3 - 511.7) | 573.7 (290.0 - 1042.2) | -0.78 |
| Arterial pH | 7.4 (7.3 - 7.4) | 7.3 (7.3 - 7.4) | 0.61 |
| Partial pressure of arterial CO_2_, mmHg | 40.5 (36 - 45.5) | 43 (38 - 50) | -0.35 |
| D-Dimer >500 ng/ml | 65 (7.5%) | 152 (17.5%) | -0.31 |
| Appearance of elevated NT-proBNP, age adjusted | 32 (3.7%) | 46 (5.3%) | -0.08 |
| Creatinine, mg/dL | 1.0 (0.8 - 1.7) | 1.5 (1.0 - 2.5) | -0.26 |
| Potassium, mEq/L | 4.1 (3.8 - 4.4) | 4.3 (3.9 - 4.7) | -0.32 |
| Sodium, mEq/L | 139.0 (135.5 - 142.0) | 138.0 (135.0 - 141.0) | 0.10 |
| Hematocrit, L/L | 31.1 (26.3 - 36.3) | 32.3 (26.9 - 37.3) | -0.17 |
| White blood cells, cells per μL | 11.3 (8.3 - 15.8) | 13.0 (8.8 - 18.3) | -0.19 |
| APACHE II score | 22 (18 - 26) | 26 (21 - 30) | -0.54 |

Patient characteristics and distribution of variables by high versus low mechanical power. High mechanical power was defined as mechanical power based on the median of the cohort (≥14.5 J/min).

*Data are expressed as frequency (prevalence in %), or median (interquartile range [25th-75th percentile]).*

Abbreviations: BMI: body mass index; COVID-19: Coronavirus disease 2019; P/F ratio: ratio of partial pressure of oxygen in arterial blood and the fraction of oxygen in the inhaled air; IBW: ideal body weight; MAP: mean arterial blood pressure; NMBA: neuromuscular blocking agents; OME: oral morphine equivalent; CO_2_: Carbindioxide; NT-proBNP: N-terminal prohormone of brain natriuretic peptide; mEq: milliequivalent; APACHE-II: Acute Physiology And Chronic Health Evaluation II.

***Table S2.* Absolute number and percentage of missing values for covariates in our study cohort.**

| **Parameter** | **Absolute number of missing values** | **Percentage of missing values** |
| --- | --- | --- |
| Temperature | 189 | 10.88 |
| Arterial O_2_ pressure | 114 | 6.56 |
| Arterial pH | 111 | 6.39 |
| Creatinine | 29 | 1.67 |
| Mean arterial pressure | 28 | 1.61 |
| Hematocrit | 16 | 0.92 |
| White blood cells | 13 | 0.75 |
| Sodium | 12 | 0.69 |
| Potassium | 12 | 0.69 |
| Respiratory rate | 1 | 0.06 |

# REFERENCES

1. von Elm E, Altman DG, Egger M, Pocock SJ, Gøtzsche PC, Vandenbroucke JP, et al. The Strengthening the Reporting of Observational Studies in Epidemiology (STROBE) statement: guidelines for reporting observational studies. PLoS Med. 2007;4:e296.

2. Benchimol EI, Smeeth L, Guttmann A, Harron K, Moher D, Petersen I, et al. The REporting of studies Conducted using Observational Routinely-collected health Data (RECORD) statement. PLoS Med. 2015;12:e1001885.

3. Marcoulides KM, Raykov T. Evaluation of Variance Inflation Factors in Regression Models Using Latent Variable Modeling Methods. Educ Psychol Meas. 2019;79:874–82.

4. Logistic Regression for Matched Case-Control Studies. Appl Logist Regres [Internet]. Hoboken, NJ, USA: John Wiley & Sons, Inc.; 2005 [cited 2022 Jul 5]. p. 223–59. Available from: https://onlinelibrary.wiley.com/doi/10.1002/0471722146.ch7

5. Serpa Neto A, Deliberato RO, Johnson AEW, Bos LD, Amorim P, Pereira SM, et al. Mechanical power of ventilation is associated with mortality in critically ill patients: an analysis of patients in two observational cohorts. Intensive Care Med. 2018;44:1914–22.

6. Faul F, Erdfelder E, Lang A-G, Buchner A. G*Power 3: a flexible statistical power analysis program for the social, behavioral, and biomedical sciences. Behav Res Methods. 2007;39:175–91.

7. Chiumello D, Gotti M, Guanziroli M, Formenti P, Umbrello M, Pasticci I, et al. Bedside calculation of mechanical power during volume- and pressure-controlled mechanical ventilation. Crit Care Lond Engl. 2020;24:417.

8. Santer P, Wachtendorf LJ, Suleiman A, Houle TT, Fassbender P, Costa EL, et al. Mechanical Power during General Anesthesia and Postoperative Respiratory Failure: A Multicenter Retrospective Cohort Study. Anesthesiology. 2022;137:41–54.

9. Knaus WA, Draper EA, Wagner DP, Zimmerman JE. APACHE II: a severity of disease classification system. Crit Care Med. 1985;13:818–29.

10. Elixhauser A, Steiner C, Harris DR, Coffey RM. Comorbidity measures for use with administrative data. Med Care. 1998;36:8–27.

11. Januzzi JL, van Kimmenade R, Lainchbury J, Bayes-Genis A, Ordonez-Llanos J, Santalo-Bel M, et al. NT-proBNP testing for diagnosis and short-term prognosis in acute destabilized heart failure: an international pooled analysis of 1256 patients: the International Collaborative of NT-proBNP Study. Eur Heart J. 2006;27:330–7.

12. Shah S, Shah K, Patel SB, Patel FS, Osman M, Velagapudi P, et al. Elevated D-Dimer Levels Are Associated With Increased Risk of Mortality in Coronavirus Disease 2019: A Systematic Review and Meta-Analysis. Cardiol Rev. 2020;28:295–302.

13. VanderWeele TJ. On the distinction between interaction and effect modification. Epidemiol Camb Mass. 2009;20:863–71.

14. Semler MW, Casey JD, Lloyd BD, Hastings PG, Hays MA, Stollings JL, et al. Oxygen-Saturation Targets for Critically Ill Adults Receiving Mechanical Ventilation. N Engl J Med. 2022;387:1759–69.

15. Yehya N, Hodgson CL, Amato MBP, Richard J-C, Brochard LJ, Mercat A, et al. Response to Ventilator Adjustments for Predicting Acute Respiratory Distress Syndrome Mortality. Driving Pressure versus Oxygenation. Ann Am Thorac Soc. 2021;18:857–64.

16. Liu D, Cui P, Zeng S, Wang S, Feng X, Xu S, et al. Risk factors for developing into critical COVID-19 patients in Wuhan, China: A multicenter, retrospective, cohort study. EClinicalMedicine. 2020;25:100471.

17. Moschovis PP, Lu M, Hayden D, Yonker LM, Lombay J, Taveras E, et al. Effect modification by age of the association between obstructive lung diseases, smoking, and COVID-19 severity. BMJ Open Respir Res. 2021;8:e001038.

18. Sunderraj A, Cho C, Cai X, Gupta S, Mehta R, Isakova T, et al. Modulation of the Association Between Age and Death by Risk Factor Burden in Critically Ill Patients With COVID-19. Crit Care Explor. 2022;4:e0755.

19. Corraini P, Olsen M, Pedersen L, Dekkers OM, Vandenbroucke JP. Effect modification, interaction and mediation: an overview of theoretical insights for clinical investigators. Clin Epidemiol. 2017;9:331–8.

20. Schuijt MTU, Schultz MJ, Paulus F, Serpa Neto A, PRoVENT–COVID Collaborative Group. Association of intensity of ventilation with 28-day mortality in COVID-19 patients with acute respiratory failure: insights from the PRoVENT-COVID study. Crit Care Lond Engl. 2021;25:283.

21. Baedorf Kassis E, Loring SH, Talmor D. Recruitment maneuvers: using transpulmonary pressure to help Goldilocks. Intensive Care Med. 2017;43:1162–3.

22. Zhang Z, Zheng B, Liu N, Ge H, Hong Y. Mechanical power normalized to predicted body weight as a predictor of mortality in patients with acute respiratory distress syndrome. Intensive Care Med. 2019;45:856–64.
